# Progenitor cell therapy for sacral pressure sore: a pilot study with a novel human chronic wound model

**DOI:** 10.1186/scrt407

**Published:** 2014-01-29

**Authors:** Reto Wettstein, Miodrag Savic, Gerhard Pierer, Oliver Scheufler, Martin Haug, Jörg Halter, Alois Gratwohl, Michael Baumberger, Dirk Johannes Schaefer, Daniel Felix Kalbermatten

**Affiliations:** 1 Department of Plastic, Reconstructive, Aesthetic and Hand Surgery, University Hospital of Basel, Spitalstrasse 21, CH-4031 Basel, Switzerland; 2 Clinic of Hematology, University Hospital of Basel, Petersgraben 4, CH-4031 Basel, Switzerland; 3 Swiss Paraplegic Center Nottwil, Guido A. Zäch-Strasse 1, CH-6207 Nottwil, Switzerland

## Abstract

**Introduction:**

Chronic wounds are a major health-care issue, but research is limited by the complexity and heterogeneity in terms of wound etiology as well as patient-related factors. A suitable animal model that replicates the situation in humans is not available. Therefore, the aim of the present work is to present a standardized human wound model and the data of a pilot study of topically applied progenitor cells in a sacral pressure sore.

**Methods:**

Three patients underwent cell harvest from the iliac crest at the time of the initial debridement. Forty-eight hours after bone marrow harvest and debridement, the CD34^+^ selected cell suspension was injected into the wound. With the aid of a laser scanner, three-dimensional analyses of wound morphometry were performed until the defect was reconstructed with a local flap 3 weeks after debridement.

**Results:**

Decreases in volume to 60% ± 6% of baseline on the sham side and to 52% ± 3% of baseline on the cell side were measured. Histologic work-up revealed no signs of metaplastic, dysplastic, or neoplastic proliferation/differentiation after progenitor cell treatment. CD34^+^ cells were detected in the biopsies of day 0.

**Conclusions:**

The pressure sore wound model allows investigation of the initial 3 weeks after cell-based therapy. Objective outcome analysis in terms of wound volume and histology can be performed without, or with, minimal additional morbidity, and the anatomy of the sacral area allows a control and study side in the same patient. Therefore, this model can serve as a standard for wound-healing studies.

**Trial registration:**

ClinicalTrials.gov NCT00535548.

## Introduction

Chronic wounds are an enormous burden for the patients and their families, represent a major challenge for the treating clinicians, and have a tremendous impact on health-care costs. A considerable amount of research has been dedicated to different types of wound dressings. The impact of these modern dressing types on wound healing, however, is frequently disappointing as their efficacy has not been validated in late-stage clinical trials. This may be related to the fact that single agents cannot interfere with the complex interplay of wound healing.

Recent interest in the treatment of chronic wounds has shifted from the type of dressing—with or without pharmaceutical agents and growth factors—to cell-based therapies. Stem cell therapies, using hematopoietic stem cells for non-hematopoietic indications as well as non-hematopoietic stem cells for tissue repairs, have increased over the last years for a broad series of indications [1]. Promising results have been reported in the treatment of small series of mainly chronic lower-extremity wounds with bone marrow-derived stem cells [2-9]. The rationale behind the use of cell-based therapies—besides the presence of macro- and microvascular disease leading to ischemia and hypoxia or hyperglycemia, infection, and inflammatory reactions and so on—is the fact that cells in chronic wounds are phenotypically altered or senescent or both [10,11]. Therefore, they have a limited capacity to divide and are less responsive to stimulation by growth factors. Also, the multimodal properties of stem and progenitor cells that create a local environment conductive to wound healing are lacking.

One of the major problems in chronic wound research is the heterogeneity of the patient population suffering from chronic wounds in terms of ulcer etiology (that is, traumatic, diabetic, venous, arterial, mixed, pressure, and radiation-induced). In addition, ulcer size and localization vary as patient comorbidities, overall health status, nutritional status, and social environment do. On the other hand, the combination of different treatment modalities, the timing and mode of their application, and the pre- and post-treatment wound care to obtain stable wound closure without recurrence—the ultimate goal—differ in many ways and render an objective analysis of any potential benefit of a treatment strategy difficult, not to mention the different sources and preparations of cells used in cell-based therapies.

Another major issue in chronic wound research, besides the heterogeneity of wound etiology and patient-related factors, is the lack of suitable animal models replicating chronic wounds. Mimicking the complexity of chronic wounds in an animal is currently not possible. This further underscores the need for a standardized human wound model. To improve these methodological limitations, a novel human chronic wound model is presented that allows a standardized, objective outcome analysis of the wound dimensions, for histological work-up and ideally for a direct comparison of two different treatment modalities in the same patient. In this pilot study, the effect of topically applied hematopoietic-derived progenitor cells on wound healing in a sacral pressure sore wound model was evaluated.

## Patients and methods

### Patients

Complete para- or tetraplegic patients who were hospitalized at the Swiss Paraplegic Center in Nottwil, Switzerland, and who presented with a primary sacral pressure sore grade III-IV (that is, without bone involvement and signs of osteomyelitis) were prospectively included in the study. Exclusion criteria were age of less than 18 or more than 50 years and presence of HIV, hepatitis B or C, active malignancy, malnutrition, diabetes mellitus, smoking, cardiopulmonary disease, peripheral arterial vascular disease, or systemic diseases like chronic polyarthritis, lupus, or scleroderma. Also, patients on steroids, chemotherapeutics, or oral anticoagulants were excluded from the study. The protocol was approved by the local institutional review board of the Canton of Lucerne (#552). Written informed consent was obtained from all patients included in the study and included approval for publication of information about themselves and fotodocumentation in this journal. Each patient signed these forms of consent.

### Wound preparation

Wound debridement was performed in a standardized en-bloc technique in the operation room under sterile conditions. Hemostasis was achieved by cauterization and application of warm compresses. For all operations, minimal anesthesia care was provided for patient monitoring. No local or systemic anesthetics were necessary in this patient group. Wound dressings during the entire study period were performed with moist gauzes (Ringer’s lactate) twice a day. No disinfectant substances were used.

### Cell harvest and progenitor cell isolation

At the time of wound debridement, bone marrow (100 mL) was harvested from the posterior iliac crest by repetitive punctures and aspirations. Cell count, CD34 count, viability, and microbiologic cultures were performed at the stem cell laboratory of the University Hospital of Basel. After overnight storage, CD34 selection was performed with magnetic beat-loaded antibodies with the CliniMACS cell separation model CS2-CE/UL (Miltenyi Biotec, Bergisch Gladbach, Germany). After selection, cell count, CD34 and lymphocyte subclass counts, viability and sterility tests were performed. Cells were once again stored overnight and then concentrated.

### Progenitor cell therapy

**Figure 1:**
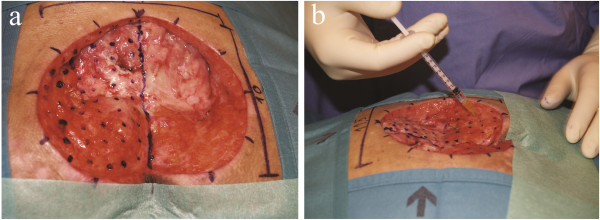
**Mapping of the pressure sore 48 hours after debridement with the planned injections sites for the stem cells on the left side. (a)** The stem cell solution is used on one side of the pressure sore, and a sham solution is used on the other side (at a distance of 1 cm in radius around injection sites). The midline can easily be identified by orientation of anatomical landmarks (rima ani, spine, or the anus). For the sake of clarity, the injection points on the control side are not included in the picture. **(b)** Injection of the progenitor cells.

Forty-eight hours after bone marrow harvest and debridement, the CD34^+^ selected stem cell suspension was injected into the wound. One side of the wound was treated by injection of the cell suspension, and the other side served as control and was treated with an identical volume of NaCl 0.9%. For injection, the wound was subdivided with a grid into small areas of 1 cm^2^ (Figure 1). Fractionated injections were performed into the wound bed as well as perifascially, subcutaneously, and subdermally at the borders of the wound.

### Endpoints

#### Wound morphometry

Changes in wound diameter and defect volume were assessed with a three-dimensional (3D) laser scanner (VI-910 Non-contact 3D digitizer; Konica Minolta Inc., Tokyo, Japan). The method has been described in detail elsewhere [12,13]. Briefly, with the patient in the prone position, the scanner was positioned parallel to the wound with a custom-made carrier that essentially consists of a C-shaped frame that permits the scanner to be positioned over the bed, at a fixed distance from the pressure sore. The captured images were imported with the Geomagic studio 12 program (Geomagic Inc., Raleigh, NC, USA) and converted into a single virtual 3D model for further analysis. Care was taken that the patient was lying flat without any sheets or pillows that may create an oblique incidence. With FreeForm modeling plus and Phantom desktop software (Sensable, Wilmington, MA, USA), the borders of the pressure sore are marked. In this procedure, in contrast to other plastic surgical procedures in which laser scanning can be used (for example, breast volume changes), definition of the borders is unequivocal in sacral pressure sores. Also, the midline of the pressure sore can be easily defined by orientation on the spine cranially and the rima ani caudally. Since there is no undermining of the wound edges after the debridement, no shadows occur. Two patches representing the two sides of the defect were created. The surface of the patches was adapted to the anatomic curvature of the sacrum. The respective dimensions of the treated and untreated sides can then be calculated and were indicated in absolute values.

#### Histology

For histological examination, two punch biopsies of 2 mm in diameter were harvested at two sites on each side of the wound on corresponding localizations at days 0 (day of stem cell injection), 3, 5, 12, and 19 (that is, at the different phases of wound healing). To avoid taking biopsies from the same location twice, a clockwise rotation was performed in predefined sectors. Biopsies were fixed in 4% buffered formaldehyde and paraffin-embedded. The slides (10 μm) were stained with hematoxylin-eosin, Giemsa, and periodic acid-Schiff staining for analysis of signs of metaplasia, dysplasia, or malignant transformation. For identification of CD34^+^ cells, an immunohistochemical reaction with intrahepatic leukocyte-4 (IHL-4) was performed. No anesthesia was required for harvesting the biopsies.

### Study protocol

In keeping with the general treatment concept of pressure sores at our institution, all wounds were debrided, conditioned, and reconstructed after 21 days [14]. At the initial debridement session, cells were harvested from the posterior iliac crest. Forty-eight hours later, the cells were injected into the wound. In all patients, the left side of the pressure sore was treated with cells. Biopsies were taken at the initial debridement and on days 0 (day of stem cell application), 3, 5, 12, and 19 after cell application. For scanning analysis, day 5 was considered the baseline value in order to eliminate any potential confounding factors such as edema by surgical trauma and local fluid irrigation. Flap coverage of the defect was performed after re-debridement and the last endpoint assessment. Clinical follow-up of 2 years after cell application was chosen for clinical monitoring for any signs of development of malignancy.

## Results

### Patients

Of a total of 35 patients presenting with sacral pressure sores between January 2007 and December 2008, three male patients met the restrictive inclusion criteria. The ages of the patients were 40, 20, and 49 years. Exclusion criteria were pressure sores of higher than grade IV in 11 cases, comorbidities in 10 cases, and absence of informed consent in three cases, and three patients had preserved sacral sensibility. Three patients were too young, two patients presented with osteomyelitis, and one had positive bacterial culture of the bone marrow aspirate. The study was finished prematurely to widen the inclusion criteria.

### Cell injection

The total numbers of injected CD34^+^ cells were 25.5 × 10^6^ (patient 1: 4.2 mL), 18.3 × 10^6^ (patient 2: 4.5 mL), and 17.4 × 10^6^ (patient 3: 3.8 mL). The values of injected CD34^+^ cell count/cm^2^ of the treated wounds were 0.5 × 10^6^, 2.2 × 10^6^, and 0.6 × 10^6^ cells/cm^2^ for patients 1, 2, and 3, respectively.

### Histology

**Figure 2:**
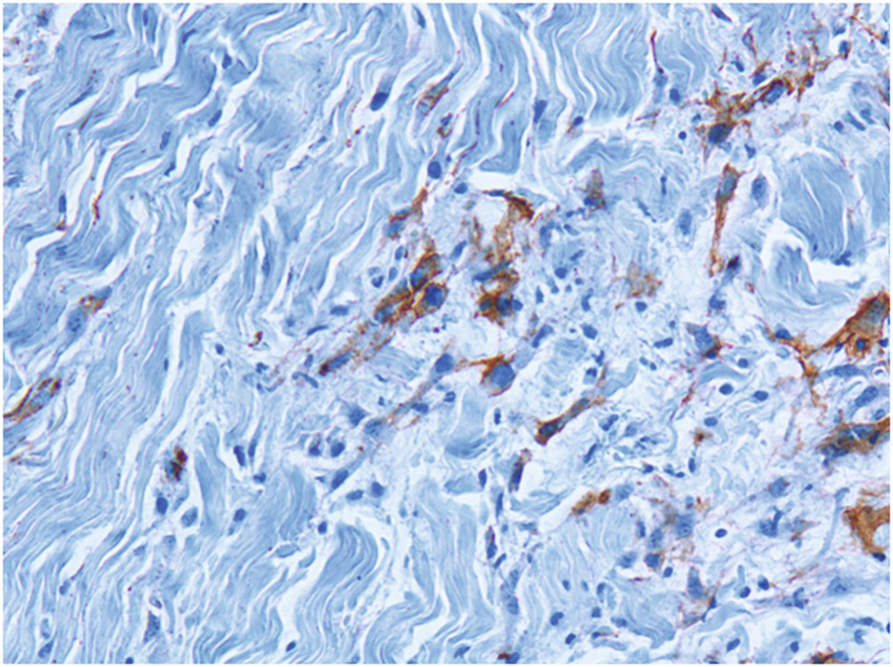
**Biopsy at day one confirming the presence of CD34**^**+ **^**progenitor cells marked in brown (immunohistochemical reaction with intrahepatic leukocyte-4 (IHL-4).** Magnification 20×.

In total, 120 biopsies were examined at the Institute of Pathology, University Hospital Basel, Switzerland. None of the biopsies showed signs of metaplastic, dysplastic, or neoplastic proliferation/differentiation after progenitor cell treatment. CD34^+^ stem cells were detected in the biopsies of day 0 (day of application) (Figure 2). From day 3 on, progenitor cell could not be identified with certainty anymore.

### Three-dimensional laser scans

**Figure 3:**
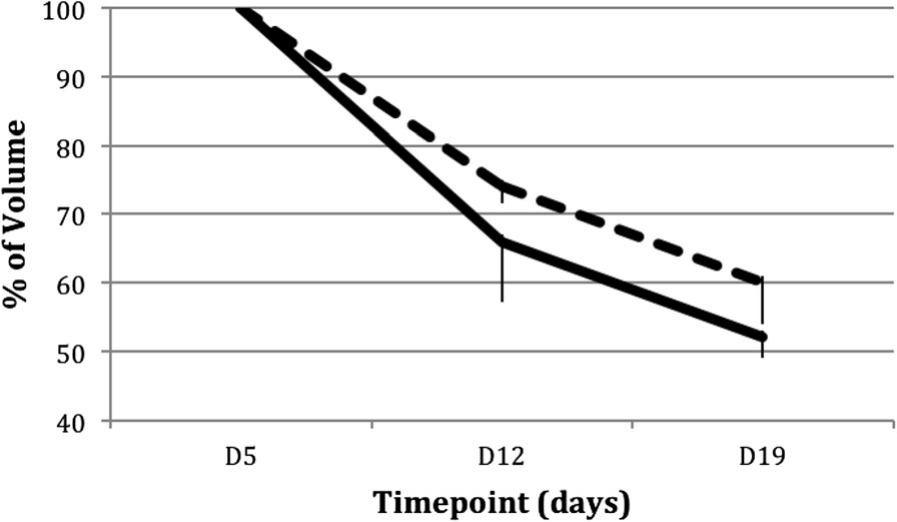
**Decrease in the volume of the wound on the control side (dotted line) and the stem cell side (continuous line) normalized to the values of day 5 (mean ± standard deviation).** D, day.

**Figure 4:**
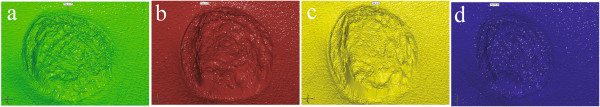
**Laser scanning images of the same pressure sore.** Image-processed scans of a treated pressure sore **(a)** after treatment and at **(b)** 5 days, **(c)** 12 days, and **(d)** 19 days.

The total volume and circumference of the treated and untreated sides of the pressure sores at day 5 are summarized in absolute values in Table 1. Within the 14 days from day 5 to day 19, there were decreases in volume to 60% ± 6% of baseline on the sham side and to 52% ± 3% of baseline on the progenitor cell side (Figure 3). Changes in the circumference of the two sides were less spectacular, with decreases to 86% ± 9% and 82% ± 4% of baseline on the sham and progenitor cell sides (Figure 4).

**Table 1:** Wound morphology data

	**Volume, cm**^**3**^		**Circumference, mm**	
	**Control side**	**Stem cell side**	**Control side**	**Stem cell side**
Patient 1	905	660	190	180
Patient 2	53	36	77	70
Patient 3	253	310	138	138

Absolute values as assessed by three-dimensional laser scanning at day 5 of the volume of the pressure sore and the circumference.

### Follow-up

Healing after flap surgery was uneventful in the three patients. During the regular follow-up examinations for 2 years post-operatively, no signs of malignancy were detected by local clinical inspection and palpation.

## Discussion

Basically, standard treatment for advanced pressure sores in plegic patients consists of debridement, wound conditioning, and pressure offload followed by defect closure with local flap procedures [14]. The time for wound bed preparation between debridement and defect closure is usually about 3 weeks. This interval represents a window for experimental investigations. Since most of sacral pressure sores cross the midline—a landmark which is easily identified and which can serve as an orientation in the analysis of scanning—each patient, respectively one of the two halves of the pressure sore can serve as control. The midline represents an easily identifiable landmark that permits to consistently divide the sacral pressure sore in a left and right half, respectively a control and treatment side. Another advantage of the flat wound appearance is the accessibility for scans. In flat wounds, compared with deep wounds, there are fewer scan shadows, thus reducing the number of scans needed to get the whole picture. In addition, it is routine practice to excise the wound borders and ground before flap closure, a manoeuver that provides local control after application of test agents. During scanning and biopsies, the patient remains in a comfortable prone position, and this increases the patient’s compliance greatly, reducing the biopsy process time and increasing the quality of the scans by reducing body movement. Another advantage of this wound model is that intermediate biopsies can be harvested without significant additional morbidity and without the need for anesthesia (in complete para-/tetraplegic patients), which may positively influence the quality of the histologic work-up.

In the three patients included in this pilot study, the single application of autologous, isolated, but unexpanded hematopoietic stem cells seems to positively influence granulation tissue formation and wound contraction as assessed by 3D laser scanning, which showed about a 50% reduction in the volume of the pressure sore on the treated side versus a 40% reduction on the control side. Obviously, the number of patients is very small and the results cannot be analyzed statistically and the biological or clinical benefit may be minimal at best; however, the aims of study were to establish this pressure sore model and to exclude any signs of malignancy after local application of cells in these three patients who were observed for 3 weeks. The number of patients and the follow-up certainly render any comment on safety and efficacy of this approach impossible. The strict inclusion criteria in this safety study restricted the number of patients included considerably. A reduction in ulcer size of more than 70% when compared with 30% in control subjects was identified after 12 weeks; however, cells were administered several times [8]. The interval for maximal action of a cell-based therapy in a previous study has shown that no changes occurred after 14 days, a time point at which minimal wound size was measured after a single cell application [15]. Fibroblasts, which are attracted from the edge of the wound or from the bone marrow, are stimulated by macrophages and some differentiate into myofibroblasts [16]. This effect might be accentuated by applying stem cells into the wound and may partly explain the trend toward increased wound contraction observed in the present study. In the present study, the amount of wound volume reduction by the formation of granulation tissue is less relevant since defect closure was performed with a flap. For most clinical cases, stimulation of granulation tissue formation, increased wound contracture and epithelialisation would be the primary goals in order to shorten the overall treatment period.

Different reports on cell-based chronic wound therapy showed a positive influence on wound healing; however, a precise and objective analysis of the effect of the therapies used is difficult, and frequently there are no data on wound contracture, development of granulation tissue, or epithelialization [3,5,7,8,15,17-19]. Some of these reports show complete healing of long-lasting chronic, usually lower extremity, wounds. However, either multiple cell applications or combinations of cell therapy and skin grafting procedures were used and led eventually to wound closure. Whereas stable wound closure is the clinically important endpoint, the pathophysiological mechanisms underlying the conversion from a chronic wound, with its derangements in the healing cascade, to a healing wound need further investigation. The sacral pressure sore model is ideal to analyze the initial period of wound changes after cell application (that is, the conversion from a non-healing to a healing wound); however, it has the obvious disadvantage that healing by secondary intention would take a long time, potentially yield unstable scar tissue at the site of pressure, and therefore be unethical. In this pilot study, no attempt was made to establish a dose dependency of the cell-based therapy, and the patients received different numbers of cells in total and per surface area treated. Obviously, in a large-scale study, the number of cells administered should be matched to the defect size.

In terms of safety, histological analysis did not reveal any signs of malignant transformation in the present study. This has been corroborated by different studies using bone marrow-derived cells for the treatment of chronic wounds, where no changes in inflammatory reactions or in differential blood count were observed or changes in laboratory analysis occurred [8,15]. The long-term clinical follow-up examinations did not show any signs of cancerous masses in the area of treatment. Also, adverse effects of the cell application have not been observed in the present study or previously reported. Although malignant transformation is an uncommon event in the chronic wound environment [20], studies showed that signaling pathways of healing skin wounds strongly resemble those of malignant tumors [21]. Our results showed that there is no sign of malignant transformation during the observed period. Furthermore, the design of the study reduces the potential risk of progenitor-cell induced malignant tissue transformation by the fact that the treated area is totally excised before surgical wound closure.

Future studies with this model will allow researchers to analyze the effect of cell preparation, selection, culture, and storage and to identify the potential of cells from other sources than bone marrow, which is a very well-known origin of hematopoietic and non-hematopoietic stem cells, including mesenchymal stem cells. Obviously, proper characterization of cells is essential in order to obtain consistent, reproducible results. Another essential parameter is the concentration of cells, since a relationship between the number of cells applied and the decrease in wound size observed has been reported [2]. Since the early inflammatory phase is characterized by migration into the wound of monocytes, which differentiate into macrophages [22] and since bone marrow-derived cells from the hematopoietic stem cell lineage increase in number in dermal wounds and lead to increased wound contraction [23], hematopoietic stem cells were used in this pilot study. Also, the harvesting of bone marrow at the time of the initial debridement did not require patient repositioning and was easy to perform. With increasing knowledge about stem cells, it seems as if different sources of cells may be used and may result in similar effects.

One of the issues in stem cell-based wound therapy is the mode of cell delivery [24]. In the present model, the cells were injected directly into the wound borders and wound ground, which was possible since pressure sores with exposed bone were not included in the study. Direct injection in the wound ground can be difficult in long-standing leg ulcers with a thick layer of dense scar tissue, which is sometimes accompanied by calcifications. Potential alternatives to direct injection are cell suspension in fibrin glue [2] and impregnation in a collagen matrix [6]. Obviously, the wound ground has to provide a critical vascularization for the cells to survive. One of the reasons to perform surgical debridement is to remove fibrin, necrotic tissue, and scar tissue to obtain a bleeding wound bed. This, however, is probably the major problem in chronic leg ulcers, in which a through debridement frequently exposes tendons or bone or a limited debridement only leads to scarce bleeding points. In such a situation, the injection of the cells is supposed to induce neoangiogenesis, which ultimately leads to the formation of granulation tissue. Cell-enhanced dressings are probably of little value in such situations. Another limitation of this study is that the patient served as his own control, and it cannot be excluded that homing of the cells applied to the saline-treated control occurred and had a positive influence on this side as well.

## Conclusions

The proper and safe use of progenitor cells, as well as the ideal source and type of cells, is currently not known. One of the reasons is the lack of a suitable model to investigate the effects of cell-based therapies on chronic wounds. The presented pressure sore wound model allowed us to investigate the initial 3 weeks after cell-based therapy and thereby to further elucidate the underlying pathophysiologic mechanisms. Since objective outcome analysis in terms of wound volume and histology can be performed with no or minimal additional morbidity, this model can serve as a standard model for wound healing. In this pilot feasibility and safety study, there was a trend toward increased granulation tissue formation and wound contracture in the three patients assessed.

## Competing interests

The authors declare that they have no competing interests.

## Authors’ contributions

MS carried out patient management, stem cell treatment, assessment of results, and histologic work-up and helped to write the manuscript. RW and DJS carried out study design and data analysis, helped to write the manuscript, but received no funding. GP and MH carried out study design, helped to revise the manuscript, but received no funding. OS carried out study design, patient management, and stem cell treatment but received no funding. JH and AG carried out study design and stem cell preparation, helped to revise the manuscript, but received no funding. MB carried out study design and patient management, helped to revise the manuscript, but received no funding. DFK carried out study design, patient management, and data analysis, helped to write the manuscript, but received no funding. All authors read and approved the final manuscript.
